# A Complex Case of Lemierre’s Syndrome With Facial Vein Involvement

**DOI:** 10.7759/cureus.23420

**Published:** 2022-03-23

**Authors:** Alexandra Cloutet, Ramya Krishna Botta, Shreedhar R Kulkarni, Pramod Kumar Ponna

**Affiliations:** 1 Department of Internal Medicine, Louisiana State University Health Sciences Center, Shreveport, USA; 2 Division of Infectious Disease, Vanderbilt University Medical Center, Nashville, USA

**Keywords:** high mortality, young population, facial vein thrombosis, internal jugular vein thrombophlebitis, lemierre's syndrome

## Abstract

Lemierre's syndrome is a rare disease that generally occurs in young, healthy individuals, where an index of suspicion for something so serious is often low. There is no standardized definition of Lemierre's syndrome, which has led to a dilemma if Lemierre's can be diagnosed without internal jugular vein (IJV) thrombophlebitis. We highlight a complex case of Lemierre's syndrome that deviates from the classical presentation of the disease. A 31-year-old male presented to the hospital with "throat swelling" and difficulty swallowing. He was in severe sepsis with end-organ damage. The patient developed severe pneumonia with pleural/pericardial effusions and bilateral nodular necrosed lesions during hospitalization. A facial vein thrombus was diagnosed, but the absence of internal jugular vein involvement initially delayed Lemierre's diagnosis. However, blood culture speciation revealed *Fusobacterium necrophorum, *which supported the suspected diagnosis. Persistent fevers and leukocytosis complicated the hospital course despite appropriate antibiotic coverage. The patient ultimately required bilateral thoracotomy and a pericardial window. He made a full recovery.

## Introduction

Lemierre's syndrome classically refers to septic thrombophlebitis of the internal jugular vein (IJV). It starts as an oropharyngeal infection classically involving *Fusobacterium*, a gram-negative anaerobe, and requires septic emboli as a diagnosis component [1]. Though this disease used to be more common before the advent of antibiotics, it is now considered rare, with an estimated incidence of 3.6 cases per 1 million per year [2]. A sore throat is the most common symptom reported, followed closely by pleuritic chest pain, dyspnea, cough, and neck/ear pain [3]. The most common physical findings are fever and pharyngitis/peritonsillar abscess [4]. Lemierre's syndrome carries a 5-18% mortality rate, making it imperative to arrive at a diagnosis promptly [5-6]. Our case involves a rarer finding that delayed the time to diagnosis. We discuss the need to broaden classic diagnostic criteria and anticoagulation recommendations in patients with Lemierre's syndrome.

## Case presentation

A 31-year-old Caucasian male presented to the hospital complaining of "throat swelling" and difficulty swallowing for seven days before admission. He tested negative for molecular group A Streptococcus and severe acute respiratory syndrome coronavirus 2 (SARS-CoV-2) antigen. Two to three days later, he developed shortness of breath and a productive cough. He developed fever and sharp right upper quadrant pain with radiation to his right shoulder. Clinically significant vital signs included a blood pressure of 90/60 mmHg, a heart rate of 120 beats/minute, and a temperature of 101.5 °F (38.6 °C). Physical examination revealed an ill-appearing young man oriented to time, place, and person. Head, ear, nose, and throat exam revealed posterior oropharyngeal erythema. No tonsillar exudate or abscesses were identified. The respiratory exam was remarkable for tachypnea, upper lobe rhonchi, and decreased breath sounds in the right lower lung field. A cardiovascular exam revealed sinus tachycardia, and the abdominal exam was remarkable for tenderness in the right upper quadrant. The rest of the physical examination was unremarkable.

Complete blood count revealed leukocytosis and thrombocytopenia. A basic metabolic panel showed hyponatremia, mild hypokalemia and hypochloremia, and increased blood urea nitrogen (BUN) and creatinine. Liver function tests revealed elevated total bilirubin, direct bilirubin, and alkaline phosphatase. Procalcitonin was elevated. The coagulation panel showed mildly elevated prothrombin time and partial thromboplastin time but significantly elevated fibrinogen (Table 1).

**Table 1:** Laboratory results at the time of admission

Test	Result (on admission)	Reference Range
White Blood Cells	19.08 K/µL	3.90-12.70 K/µL
Platelets	87 K/µL	150-450 K/µL
Sodium	130 Mmol/L	136-145 Mmol/L
Potassium	3.4 Mmol/L	3.5-5.1 Mmol/L
Chloride	93 Mmol/L	95-110 Mmol/L
Blood Urea Nitrogen	33 Mg/dl	6-20 Mg/dl
Creatinine	3 Mg/dl	0.5-1.4 Mg/dl
Aspartate Aminotransferase	34 U/L	10-40 U/L
Alanine Aminotransferase	30 U/L	10-44 U/L
Direct Bilirubin	3.7 Mg/dl	0.1-0.3 Mg/dl
Total Bilirubin	5.2 Mg/dl	0.1-1.0 Mg/dl
Alkaline Phosphatase	417 U/L	55-135 U/L
Procalcitonin	40.50 ng/ml	<0.50 Ng/ml
Prothrombin Time	15.6 Seconds	9.4-12.5 Seconds
Partial Thromboplastin Time	38.1 Seconds	25.1-36.5 Seconds
Fibrinogen	666 Mg/dl	214-454 Mg/dl

**Figure 1:**
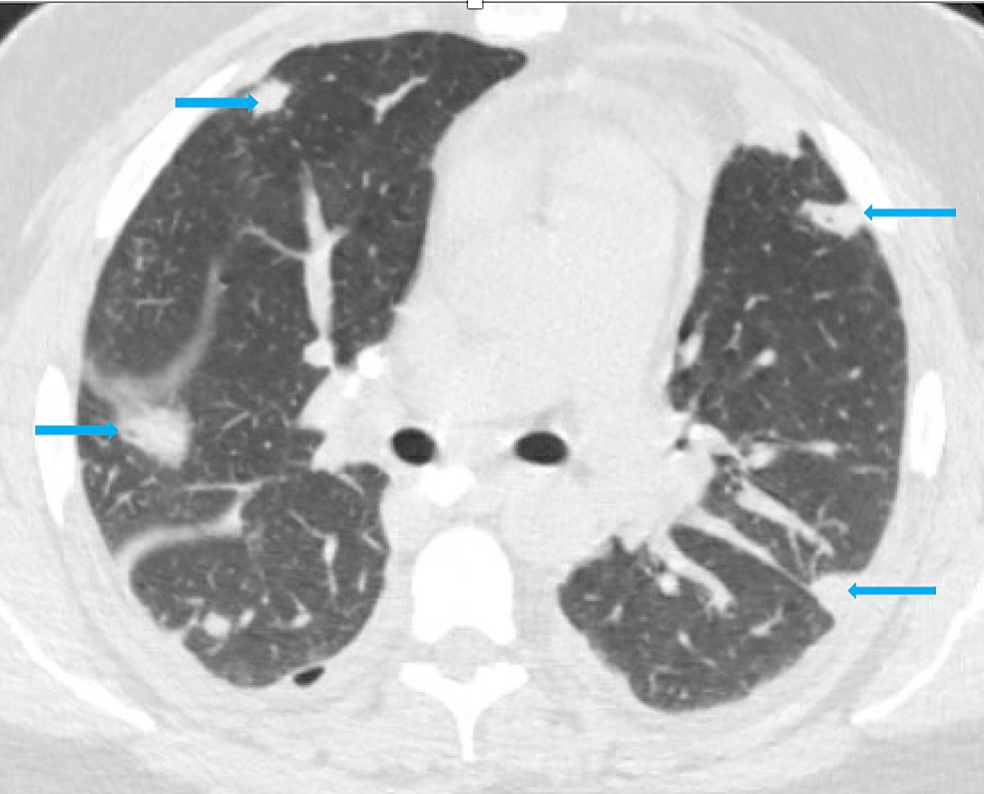
CT chest showing multiple scattered pleural nodules, some with cavitations suggestive of septic pulmonary emboli (blue arrows)

**Figure 2:**
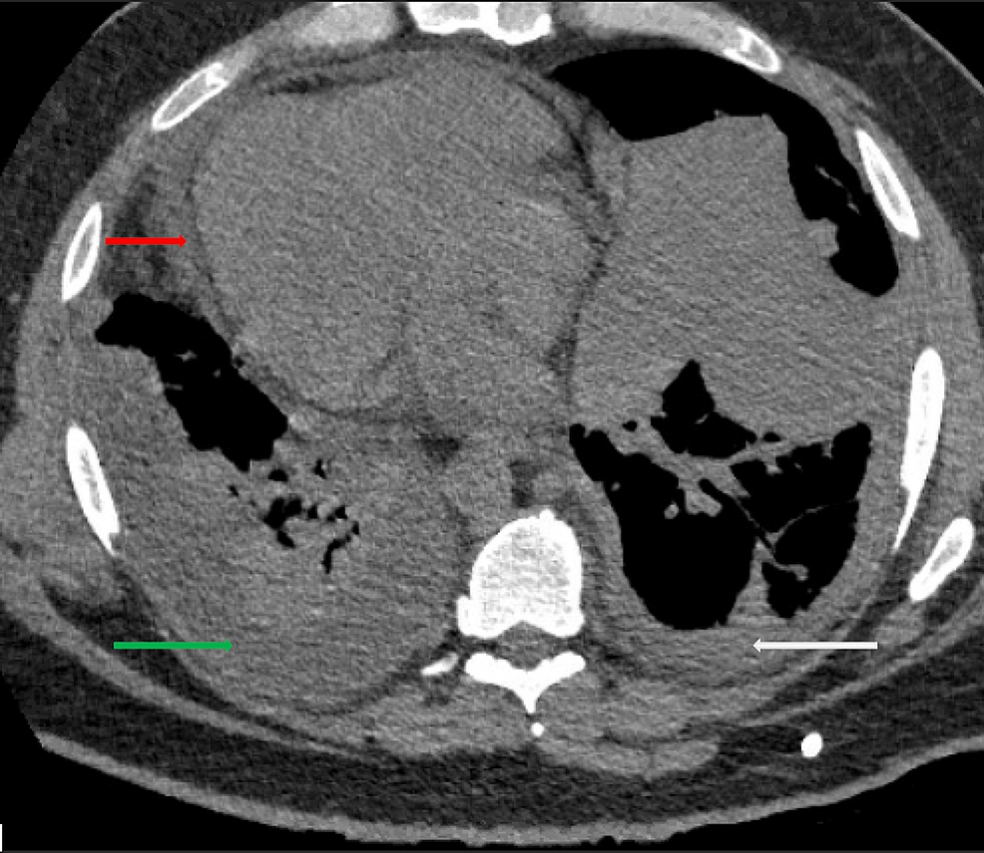
CT chest showing moderate pericardial effusion (red arrow), moderate right-sided pleural effusion (green arrow), and mild left-sided pleural effusion (white arrow)

Chest X-ray showed right lung base consolidation and an ipsilateral pleural effusion. Ultrasound abdomen was unremarkable. Computed tomography (CT) chest was remarkable for multiple scattered pleural nodules, some appearing cavitary, suggestive of septic pulmonary emboli (Figure 1), moderate right-sided pleural effusion, and a moderate pericardial effusion (Figure 2). CT soft tissue and neck revealed complicated tonsillitis with thrombosis of the left facial vein. A transesophageal echocardiogram showed a mobile echo density of 1.79 x 0.71 cm, attached to the posterior wall of the main pulmonary artery above the pulmonary valve, which was suspicious for possible vegetation. Cardiac CT revealed high density in some parts of the pericardial effusion that raised the possibility of an exudative pericardial effusion and bilateral pleural effusions with noted right-sided loculation. No valvular vegetations were visualized. Two sets of blood cultures were collected on the day of admission. They grew* Fusobacterium necrophorum* one day after admission.

**Figure 3:**
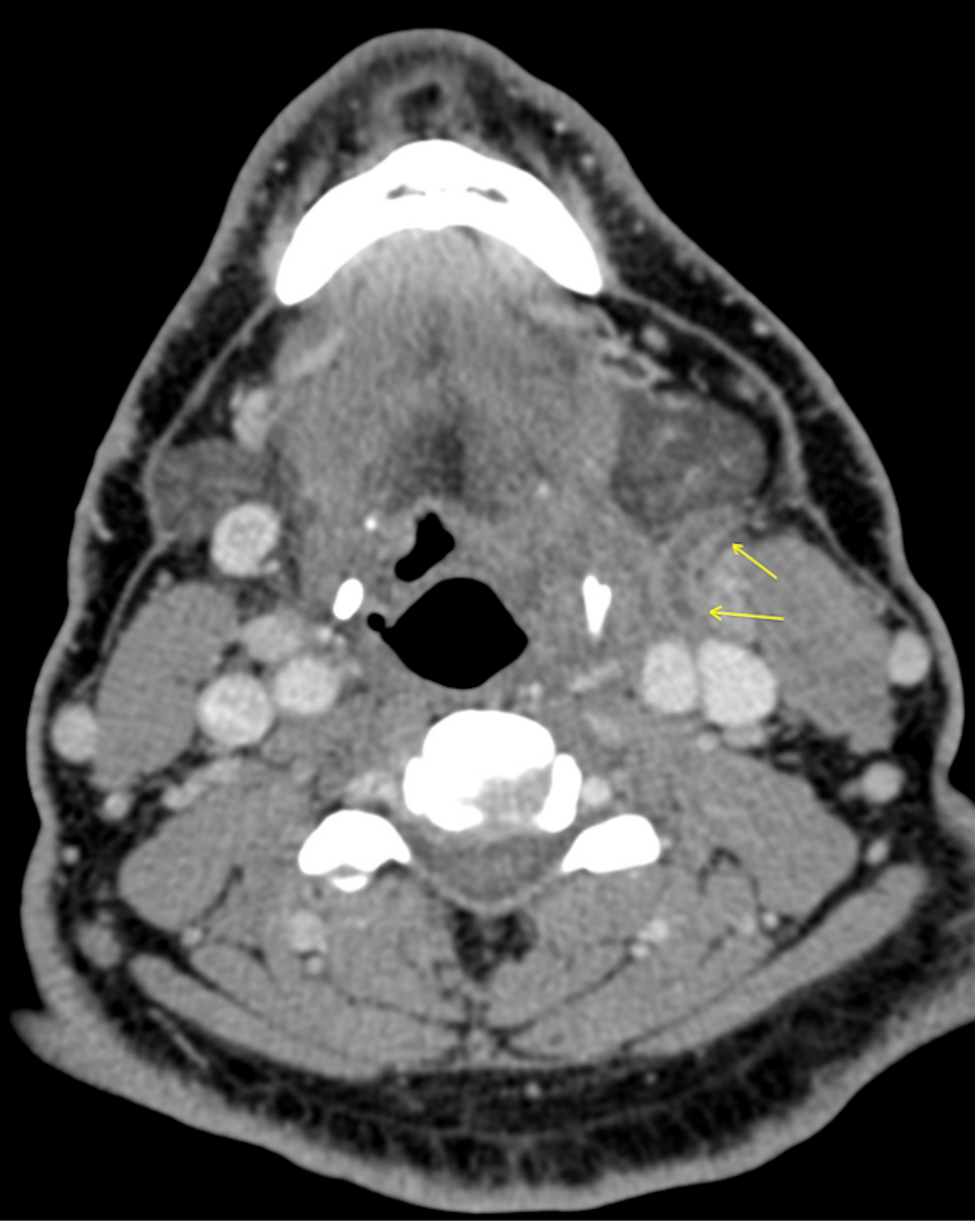
CT soft tissue neck with contrast showing with left facial vein thrombosis (yellow arrow)

The two top differential diagnoses were Lemierre’s syndrome and right-sided infective endocarditis. Bilateral lung lesions possibly due to septic emboli raised concerns for infective endocarditis. A transthoracic echocardiogram, transesophageal echocardiogram, and a cardiac CT were performed to rule out a diagnosis of right-sided infective endocarditis. They could not show conclusive evidence of vegetation, though the pulmonic valve was not well-visualized. A CT neck soft tissue was done, which revealed thrombosis of the facial vein (Figure 3) but no involvement of the internal jugular vein. However, blood cultures grew* Fusobacterium necrophorum*, validating Lemierre’s syndrome as the most likely diagnosis.

On the day of admission, the patient was empirically started on the broad-spectrum antibiotics vancomycin and meropenem to cover methicillin-resistant staphylococcus aureus (MRSA), gram-positive, gram-negative, and anaerobic pathogens. Right upper quadrant tenderness was attributed to right-sided pneumonia and pleural effusion. Repeat blood cultures performed a couple of days later were negative. Pulmonology was consulted for thoracentesis for complicated pneumonia/parapneumonic effusion. A chest tube was placed, which drained frank pus. The patient received three doses of intrapleural fibrinolytic treatment with alteplase (tPA) and dornase alfa (Dnase). Due to the non-resolution of pleural effusions, cardiothoracic surgery decided to perform a surgical intervention. The patient underwent left anterior thoracotomy with a pericardial window and drain placement and a right video-assisted thoracoscopic surgery converted to right open thoracotomy with hematoma evacuation and decortication. Bilateral chest tubes were placed at the time of surgery and removed seven days later. The pericardial drain was removed nine days later. Once the patient showed signs of clinical improvement, broad-spectrum antibiotics were discontinued and discharged on oral metronidazole for two weeks. The patient was not started on anticoagulation for facial vein thrombosis.

## Discussion

We report an atypical case of Lemierre’s syndrome with facial vein thrombus, rather than the usual IJV thrombus. While IJV thrombosis has long been considered one of the primary diagnostic criteria for Lemierre’s syndrome, many well-accepted diagnostic guidelines exclude this criterion [7]. More recent criteria require a diagnosis based on a recent oropharyngeal (or head-neck) infection; head-neck thrombosis of the internal jugular vein, other veins, or typical septic emboli; and blood cultures positive for *Fusobacterium necrophorum* or other bacteria possibly associated with the syndrome [7-8]. Some diagnostic guidelines also consider thrombosis of the IJV or any of its tributaries (including the facial vein) an acceptable criterion. There are other reported cases like ours where the thrombus was in the facial vein rather than the IJV [9-10]. A retrospective study of 11 cases of Lemierre’s syndrome found that 4/11 had thrombosis in the IJV tributaries [11].

IJV thrombosis occurs via direct bacterial spread to the parapharyngeal space or through the lymphatic system. Another proposed mechanism of spread involves an anterograde progression of thrombophlebitis from the peritonsillar venous plexus. This mechanism can serve as an explanation for facial vein thrombosis due to its connection with the peritonsillar vein [11]. This mechanism would similarly explain some cases of upward extension of the thrombosis to the intracranial veins and downward extension to the anterior jugular and external jugular veins [12]. A recently published meta-analysis of 712 Lemierre’s patients found that 16.3% presented with head/neck venous involvement other than the internal jugular, external jugular, or cerebral veins [13]. These presentations of Lemierre’s syndrome are seemingly rarer, contributing to why thrombosis of veins other than the IJV has not been incorporated into many diagnostic criteria. Cases such as ours suggest that other locations of thrombosis may need to be accepted as criteria for diagnosing Lemierre’s syndrome.

While treating this patient, there was a discussion regarding the administration of anticoagulant therapy in addition to broad-spectrum antibiotics. In the past, it was thought that anticoagulation improved outcomes for certain sequelae of Lemierre’s syndrome, including septic thromboembolic events that could lead to respiratory failure or septic arthritis [14-15]. However, literature has suggested that anticoagulation serves no role in treating classic presentations of this disease because it was thought that it did not improve thrombosis outcomes [16-17]. Additionally, a meta-analysis of 359 Lemierre’s syndrome studies with a total of 394 patients concluded that there was no significant improvement in vessel recanalization or mortality in patients treated with various anticoagulation regimens [18]. A more recent meta-analysis of 712 Lemierre’s syndrome patients suggests that anticoagulation cannot be used in patients with a high risk of bleeding or septic complications. In patients with an uncomplicated course (low bleeding risk without new septic complications), after a routine risk assessment, therapeutic dose anticoagulation could be considered. However, this meta-analysis also concluded that there was insufficient data to analyze which anticoagulant treatments were better suited to Lemierre’s patients [13]. Our team elected not to proceed with anticoagulation due to the increased risk of bleeding and possible fragmentation of the septic thrombus. Also, available literature did not show which type and duration of anticoagulation would support a mortality benefit, which is a potential area for future research to explore.

## Conclusions

Lemierre’s syndrome may be more prevalent than originally thought due to inconsistent diagnostic guidelines. It is a spectrum of clinical findings, and not all need to be present to make a diagnosis. The absence of internal jugular vein thrombophlebitis does not rule out the diagnosis. Thrombosis of other veins in the appropriate clinical setting may be needed to be included in the diagnostic criteria. More trials are required to establish safety and mortality benefits with anticoagulation in patients with Lemierre’s syndrome.
